# Warming appears as the main risk of non-adaptedness for western Mediterranean relict fir forests under expected climate change scenarios

**DOI:** 10.3389/fpls.2023.1155441

**Published:** 2023-08-11

**Authors:** Belén Méndez-Cea, Isabel García-García, Juan Carlos Linares, Francisco Javier Gallego

**Affiliations:** ^1^Dpto. Genética, Fisiología y Microbiología, Unidad Docente de Genética, Facultad de CC Biológicas, Universidad Complutense de Madrid, Madrid, Spain; ^2^Dpto. Sistemas Físicos, Químicos y Naturales, Universidad Pablo de Olavide, Sevilla, Spain

**Keywords:** circum-Mediterranean firs, selection signature, genotype-environment associations, risk of non-adaptedness, Sierra de las Nieves, Grazalema, Talassemtane

## Abstract

Circum-Mediterranean firs are considered among the most drought-sensitive species to climate change. Understanding the genetic basis of trees’ adaptive capacity and intra-specific variability to drought avoidance is mandatory to define conservation measures, thus potentially preventing their extinction. We focus here on *Abies pinsapo* and *Abies marocana*, both relict tree species, endemic from south Spain and north Morocco, respectively. A total of 607 samples were collected from eight nuclei: six from Spanish fir and two from Moroccan fir. A genotyping by sequencing technique called double digestion restriction site-associated DNA sequencing (ddRAD-seq) was performed to obtain a genetic matrix based on single-nucleotide polymorphisms (SNPs). This matrix was utilized to study the genetic structure of *A. pinsapo* populations and to carry out selection signature studies. In order to understand how Spanish fir and Moroccan fir cope with climate change, genotype–environment associations (GEAs) were identified. Further, the vulnerability of these species to climate variations was estimated by the risk of non-adaptedness (RONA). The filtering of the *de novo* assembly of *A. pinsapo* provided 3,982 SNPs from 504 out of 509 trees sequenced. Principal component analysis (PCA) genetically separated Grazalema from the rest of the Spanish populations. However, F_ST_ values showed significant differences among the sampling points. We found 51 *loci* potentially under selection. Homolog sequences were found for some proteins related to abiotic stress response, such as dehydration-responsive element binding transcription factor, regulation of abscisic acid signaling, and methylation pathway. A total of 15 associations with 11 different *loci* were observed in the GEA studies, with the maximum temperature of the warmest month being the variable with the highest number of associated *loci*. This temperature sensitivity was also supported by the risk of non-adaptedness, which yielded a higher risk for both *A. pinsapo* and *A. marocana* under the high emission scenario (Representative Concentration Pathway (RCP) 8.5). This study sheds light on the response to climate change of these two endemic species.

## 1 Introduction

The frequency and intensity of drought events are increasing worldwide, triggering extensive growth decline, dieback, and mortality in many forest ecosystems (DeSoto et al., 2020; McDowell et al., 2020). At a regional scale, the Mediterranean basin is highly vulnerable to ongoing warming, a trend that is predicted to worsen (Ozturk et al., 2015). Consequently, drought-sensitive forests are likely to be increasingly stressed, as water shortage impairs the functioning of trees by reducing their photosynthesis and growth rates (Choat et al., 2018). The circum-Mediterranean firs can be considered among the most sensitive tree species to climate change (Aussenac, 2002; Linares, 2011; Caudullo and Tinner, 2016; Sánchez-Salguero et al., 2017). Furthermore, they are mainly represented by relict tree species, which causes several uncertainties concerning their future prospects (Hampe and Jump, 2011). Ecological and evolutionary factors determine the population dynamics of these endangered tree species, while several underlying genetic responses remain elusive to our knowledge (Alberto et al., 2013). Thus, a proper understanding of key physiological and genetic mechanisms involved in the populations’ persistence becomes imperative for maintaining forest biodiversity and ecosystem services (Anderegg et al., 2013).

Here, we focus on *Abies marocana* Trab. and *Abies pinsapo* Boiss., the westernmost circum-Mediterranean fir species, endemic from north Morocco and south Spain, respectively (Linares, 2011; Ben-Said, 2022). Both species are included in the International Union for Conservation of Nature (IUCN) Red List of Threatened Species as endangered species, with climate change one of the main threats to their conservation (Alaoui et al., 2011; Arista et al., 2011). Events of forest dieback and mortality have been mainly reported for *A. pinsapo* (Linares et al., 2009; Navarro-Cerrillo et al., 2020a; Navarro-Cerrillo et al., 2022; Cortés-Molino et al., 2023), while the sensitivity to climate change of *A. marocana* has been also pointed out (Esteban et al., 2010; Navarro-Cerrillo et al., 2020b; Alaoui et al., 2021). Despite the increasing concern regarding the apparent rising mortality rates of these relict tree species in the context of pervasive environmental changes and habitat loss, the genetic mechanisms underlying contrasting vulnerability among individuals are still poorly understood. However, some efforts have been made to shed light on this issue (e.g., Cobo-Simón et al., 2021; Cobo-Simón et al., 2023b). Furthermore, a better understanding of the climatic factors affecting species’ vulnerability, such as the risk of non-adaptedness, may allow for better predictions of the success of species to track changing climates (Rellstab et al., 2016; Pina-Martins et al., 2019).

Previous genetic studies performed in *A. pinsapo* and *A. marocana* focused mainly on molecular markers of the nucleus and cytoplasm, namely, chloroplast and mitochondria, but not on the entire genome. The main reasons are related to the intrinsic characteristics of the conifer genomes, characterized by their large size (18–20 Gbp), a high number of repetitive sequences, and the absence of reference genomes for most species (Neale and Wheeler, 2019; García-García et al, 2022). Hence, there are studies determining genetic diversity and population structure using chloroplast microsatellites (cpSSRs) (Terrab et al., 2007); developing new molecular markers such as nuclear microsatellites (nSSRs) (Sánchez-Robles et al., 2012); describing genetic differentiation between *A. pinsapo* and *Abies alba* using SSRs (Jaramillo-Correa et al., 2010; Dering et al., 2014); determining the genetic diversity of several Spanish fir populations using SSRs, intermicrosatellites (ISSRs), and single-nucleotide polymorphisms (SNPs) (Cobo-Simón et al., 2020); characterizing drought response genes of *A. pinsapo* applying SNPs (Cobo-Simón et al., 2021; Cobo-Simón et al., 2023a); or performing biogeographic studies with several species of *Abies* taxa (Litkowiec et al., 2021). Nonetheless, current advances in next-generation sequencing (NGS) and in bioinformatics open a wide range of possibilities to perform studies with conifer genomes. Furthermore, transcriptomes can be used as an alternative reference. Hence, in the case of *A. pinsapo*, Pérez-González et al. (2018) obtained a *de novo* transcriptome, and recently, Ortigosa et al. (2022) published other transcriptome with the aim to determine intraspecific variations.

In this work, a genotyping by sequencing (GBS) technique named double digestion restriction site-associated DNA sequencing (ddRAD-seq) was used to obtain a genetic matrix based on SNPs. Our specific aims were i) to study the genetic structure of *A. marocana* and *A. pinsapo* populations and to determine the presence of loci under selection based on the obtained SNPs, ii) to identify genotype–environment associations (GEAs) based on local climate variables and population genetics, and iii) to assess the vulnerability of *A. marocana* and *A. pinsapo* to future climate change scenarios based on the risk of non-adaptedness (RONA).

## 2 Materials and methods

### 2.1 Species of study and field sampling

*A. pinsapo* is a sub-dioicous fir with a pyramidal shape, which can grow up to 30 m. Spanish fir is located on north-facing slopes between 1,000 and 1,800 m a.s.l. (Linares et al., 2009). This location allows *A. pinsapo* to maintain optimum conditions of humidity, which is the most relevant issue to this species’ survival. Spanish fir is a relict species whose distribution is restricted to the South of Spain. There are three nuclei, which are placed in Sierra de las Nieves with an area of approximately 5,800 ha, Sierra Bermeja with 1,212 ha, and Sierra de Grazalema with approximately 2,000 ha.

**Figure 1:**
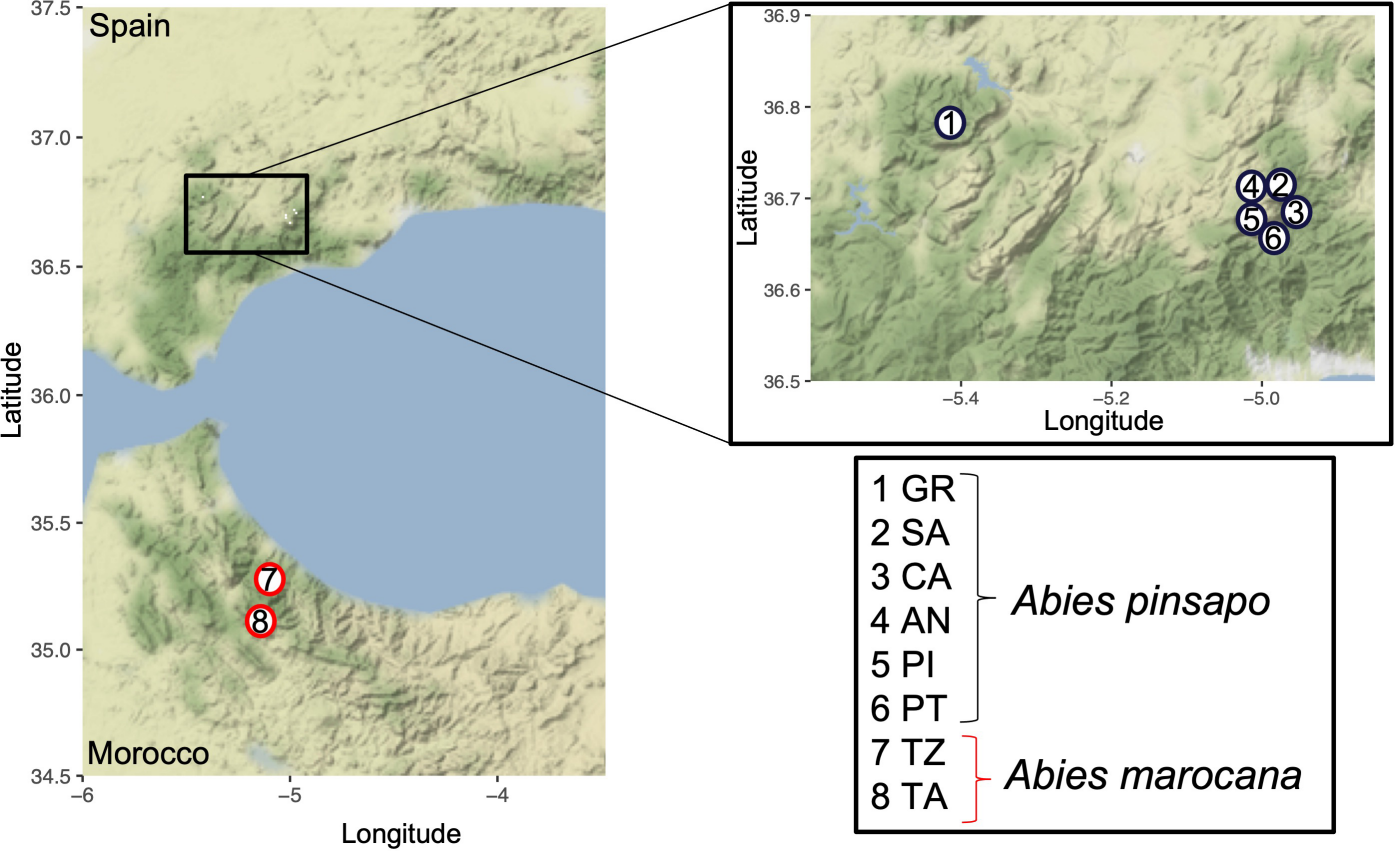
Geographical localization of the nuclei used to carry out the genetic structure of *Abies pinsapo* populations. In addition, the map shows the two places occupied by *Abies marocana*.

A total of 571 leaf samples from six populations of *A. pinsapo* were collected (**Figure 1**). The studied populations located in the National Park of Sierra de las Nieves were Saucillo (SA), with 126 samples collected; Caucón (CA), with 259 samples; Ánimas (AN), with 51 samples; Pilones (PI), with 29 samples; and Pilar de Tólox (PT), with 16. Additionally, 90 samples were sampled from the Biosphere Reserve Sierra de Grazalema (GR). Not all the samples collected were sequenced (**Table 1**). To assess the genetic differences between saplings and old trees, individuals of these two cohorts were collected from three nuclei: SA, CA, and AN (**Table 1**).

**Table 1:** Characteristics of each nucleus of study.

Species	Nucleus	Country	Abbreviation	Latitude (°N)	Longitude (°W)	Elevation (m a.s.l.)	Mean annual temperature (°C)	Mean annual precipitation (mm)	Population (n)	Saplings (n)		Old trees (n)
*Abies pinsapo*	Caucón	Spain	CA	36.71	−4.96	1,114	12.0	975.0	226	24		25
	Saucillo	Spain	SA	36.72	−4.98	1,352	11.2	1,261.6	110	12		12
	Grazalema	Spain	GR	36.78	−5.40	1,085	12.0	1,277.5	79	−		−
	Pilar de Tólox	Spain	PT	36.68	−5.00	1,669	10.2	1,647.6	16	−		−
	Pilones	Spain	PI	36.69	−5.02	1,700	10.3	1,659.3	28	−		−
	Ánimas	Spain	AN	36.70	−5.01	1,637	10.3	1,616.6	50	18		17
*Abies marocana*	Talassemtane	Morocco	TA	35.17	−5.18	1,694	10.1	1,821.0	53	23		30
	Tazaot	Morocco	TZ	35.23	−5.10	1,677	10.5	1,644.3	45	26		19

It is indicated which species is located in each region, the name of the area, their localization (latitude and longitude), elevation, and mean annual temperature and precipitation. The last three columns are related to the total number of samples genotyped with ddRAD-seq and the number of saplings and old trees genotyped of those populations in which two ages were collected.

ddRAD-seq, double digestion restriction site-associated DNA sequencing.

*A. marocana* is a monoecious species that appears in the high peaks between 1,500 and 2,000 m a.s.l. (Ben-Said, 2022). As it was described for Spanish fir, this species is usually present in the north slope because it needs humid environments to survive due to its drought sensitivity (Aussenac, 2002). Moroccan fir is a relict species whose range distribution is in Northern Morocco.

In terms of *A. marocana*, two populations located in Talassemtane National Park were studied. Talassemtane (TA) accounts for approximately 2,000 ha, and Jebel Tazaot (TZ), which is the mountain range of Talassemtane, is approximately 1,000 ha (Ben-Said, 2022; Ben-Said et al., 2022). The number of collected samples was 101, with 53 from TA and 45 from TZ (**Figure 1**). As it was described for *A. pinsapo* populations, saplings and old trees were collected from these two populations (**Table 1**).

### 2.2 DNA extraction and ddRAD-seq

The extraction starts with the lyophilization of 100 mg of each fresh leaf sample. Then, total DNA extraction was carried out by using DNeasy Plant Mini Kit (Qiagen^®^, Berlin, Germany) following the manufacturer’s instructions with some modifications. DNA concentration was measured on a NanoDrop™ spectrophotometer, and the integrity of the extracted samples was determined by electrophoresis in 1% agarose gel. Only those samples that reached the requirements for the ddRAD-seq technique (Peterson et al., 2012) were selected for genotyping (**Table 1**). In this case, a total of 509 samples of Spanish fir and 98 of Moroccan fir were optimum for the technique. Subsequently, ddRAD-seq (Peterson et al., 2012) libraries were constructed using *Ape*KI/*Pst*I double digestion and sequenced by LGC Genomics (Berlin, Germany). To know more about the methodology, please see Méndez-Cea et al. (2023a).

Due to the lack of Spanish and Moroccan firs reference genomes, a *de novo* assembly of the obtained paired-end sequences was performed. However, there is an available transcriptome of *A. pinsapo*, which allowed us to carry out a reference assembly. This transcriptome was previously obtained by our group (for more details, please see Pérez-González et al., 2018). Both assemblies and the SNP callings were performed using ipyrad v.0.9.65 (Eaton and Overcast, 2020). Several filtering steps were carried out using VCFtools v0.1.16 (Danecek et al., 2011). With the aim to retain only quality biallelic SNPs, the following parameters were used: a minor allele frequency (MAF) of 5% and maximum missingness of 50%, and, to avoid linkage disequilibrium, only one SNP per locus was maintained. Moreover, a final step removing those individuals with information for less than 50% of the filtered SNPs was performed. Several genetic matrixes were obtained depending on the dataset used in the filtration steps.

**Figure 2:**
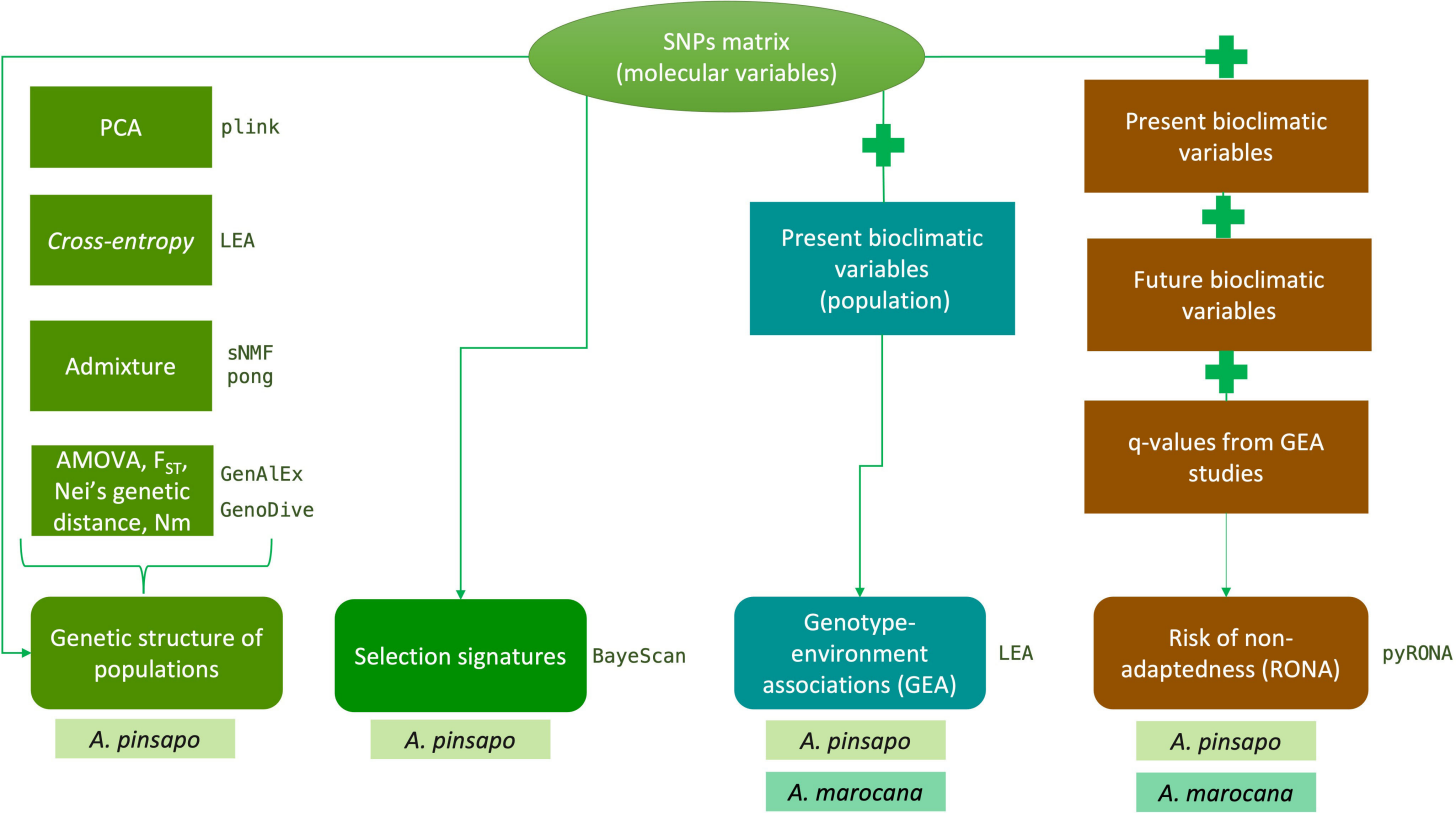
Description of the workflow followed to perform the different studies. The species used for each analysis are indicated.

The following sections are summarized as a workflow in **Figure 2**.

### 2.3 Genetic structure of populations

Two approaches were used to study the genetic structure of *A. pinsapo* populations: principal component analysis (PCA) and sparse non-negative matrix factorization (sNMF) analysis. PCA was performed to describe the inter- and intrapopulation structures, while sNMF was used to estimate ancestry coefficients (Frichot et al., 2014). PCA was carried out using the plink2 2.00a2.3 software (Chang et al., 2015) with the ––pca option, and then a graphic representation was obtained using the R v4.1.2 (R Core Team, 2022) package called ggplot2 v3.3.5 (Wickham, 2016). For the second approach, a cross-entropy study was carried out using the snmf function of the LEA package v3.4.0 (Frichot and François, 2015) in R to determine the most probable number of ancestry populations (K) that best explains the structure of the populations. In addition, the admixture coefficients were calculated too. For this analysis, a total of 10 repetitions for each K from 1 to 8 was performed. Then, a graphic representation of the admixture results was performed using the pong software package (Behr et al., 2016).

Several statistical analyses of each genetic matrix were performed using GenAlEx v6.5 (Peakall and Smouse, 2006; Peakall and Smouse, 2012) and GenoDive v3.06 (Meirmans, 2020). Parameters such as fixation indexes (F_ST_), to determine population differentiation, and migration rate (Nm), to describe the presence or not of gene flow among populations, were estimated. Nei’s genetic distance among populations was also determined. Heterozygosity, private alleles, and polymorphic loci were obtained too. Finally, the Shannon index was calculated to infer genetic diversity.

In addition, a molecular variance analysis (AMOVA) with 9,999 permutations was carried out with the aim to determine the proportion of genetic variation attributable to differences among and within populations.

### 2.4 Selection signatures

BayeScan 2.1 software (Foll and Gaggiotti, 2008) was used to identify selection signatures. Default parameters were applied. For more details, please see Méndez-Cea et al. (2023a). The significance threshold was established at 5%. The complete sequence containing the significative SNPs was queried against the nucleotide database and, in the case of the matrixes obtained from reference assembly, against the transcriptome shotgun assembly (TSA) database using, in both cases, BLASTn (National Center for Biotechnology Information (NCBI); Altschul et al., 1990). When a match was obtained, a BLASTx (NCBI; Gish and States, 1993) against the non-redundant protein database was performed too.

### 2.5 Genotype–environment associations

The 19 bioclimatic variables from the WorldClim database (Fick and Hijmans, 2017) with a resolution of 30 s were used to identify GEAs. These variables were obtained using the freeware QGIS 3.18 (Quantum Geographic Information System) (QGIS Development Team, 2022) for each location. Highly correlated variables were removed based on their Pearson’s correlation values (r > 0.9), which were calculated with the Hmisc package v4.7-0 (Frank and Harrell, 2022) in R. Since this correlation needs a minimum of four populations to be estimated, the coefficient could be obtained depending on the used genetic matrix. On the other hand, an imputation step was conducted by using the LEA R package v3.4.0 (Frichot and François, 2015) to fill in all the missing data of the genetic matrix before performing the GEA study.

The lfmm (latent factor mixed models) function of the LEA R package v3.4.0 (Frichot and François, 2015) was used to perform GEA studies. Each run was repeated 20 times with 100,000 iterations and a burn-in of 50,000. The used K value was the one obtained from the cross-entropy analysis. The p-value results were corrected to q-values using a false discovery rate (FDR), and the significance threshold was set to 5%. The complete sequences, which include the SNPs that showed significant associations with one or more bioclimatic variables, were queried against the nucleotide and protein NCBI database as it was described above.

### 2.6 Risk of non-adaptedness

The RONA value indicates the theoretical percentage of change in allele frequency at loci associated with environmental variables, which is required for a given population to cope with future changes in that variable, allowing it to survive (Rellstab et al., 2016; Pina-Martins et al., 2019). The estimation of this value is based on the average absolute difference of the changes in allele frequencies between the current and future climate conditions of those loci, which showed associations with environment variables. Low values of RONA predict a greater predisposition to adapt to new environmental conditions.

pyRona v0.3.6 (Pina-Martins et al., 2019) for lfmm results was used to calculate the RONA value at the population level. Two different climate scenarios, which are predicted to take place by the end of this century (2081–2100), were studied: low emissions (Representative Concentration Pathway (RCP) 2.6), which limits the increase of global mean temperature to 2°C, and high emissions (RCP8.5), whose limitation is 4.9°C. A total of 19 bioclimatic variables of each scenario were tested. These variables were downloaded from the WorldClim database as it was described in the GEA section.

## 3 Results

### 3.1 Genetic structure of *A. pinsapo* populations

**Figure 3:**
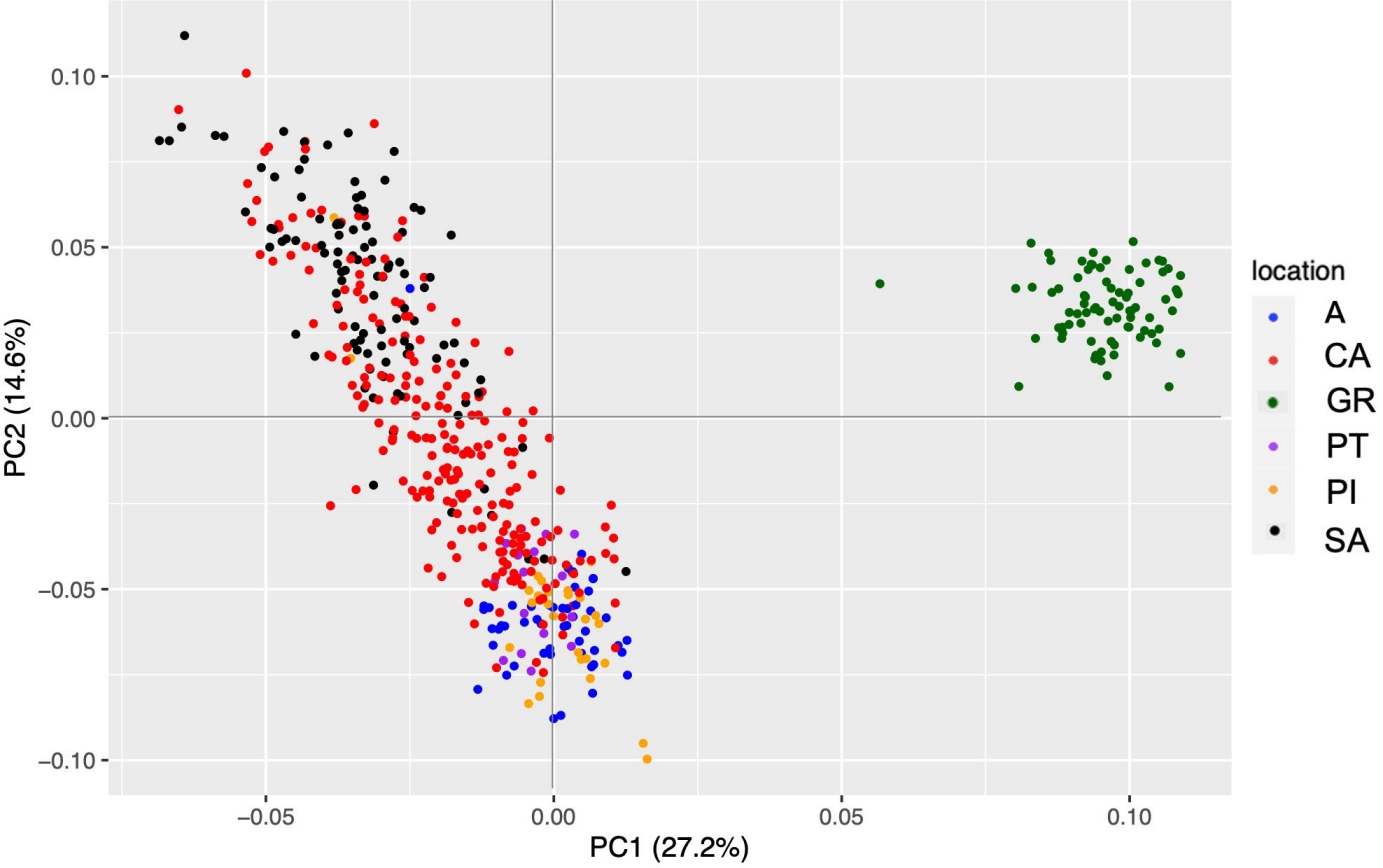
PCA representation with the *de novo* assembly dataset of *Abies pinsapo* populations. Each of them has a different color, which is indicated in the legend. PCA, principal component analysis.

The filtering of the *de novo* assembly of the Spanish fir nucleus dataset maintained 504 individuals and 3,982 SNPs. The first axis of PCA separated GR from the rest of the Spanish populations with an explanation percentage of 27.2% (**Figure 3**). AN, PI, and PT are very similar, while CA shows a gradient ranging from SA to the rest of the populations. The cross-entropy analysis, which was carried out for K values ranging from 1 to 8, did not provide much information, as the lowest obtained value was 8. However, the graphical representation of the admixture results showed that K = 3 seemed to be the best explanation for our results. In this case, GR showed its own gene pool again, SA and CA composed the second group, and the rest of the Spanish populations were grouped.

The AMOVA results of *A. pinsapo* nuclei showed the greatest differences within individuals (55.2%) and the lowest among individuals (4.3%) with a high significance (p-value = 0.000). In addition, significant differences among populations were found (40.5%).

Pairwise population F_ST_ values ranged from 0.007 to 0.062, with high significance in all the cases (p-values = 0.000), indicating the presence of genetic differences among all nucleus pairs (**Table 2**).

**Table 2:** Pairwise population F_ST_ obtained from the *Abies pinsapo de novo* assembly dataset.

Pairwise population F_ST_ values							
CA	SA	GR	PT	AN	PI		
**0.000**						CA	
0.007*	**0.000**					SA	
0.052*	0.062*	**0.000**				GR	
0.018*	0.034*	0.063*	**0.000**			PT	
0.020*	0.031*	0.059*	0.027*	**0.000**		AN	
0.020*	0.032*	0.059*	0.024*	0.016*	**0.000**	PI	

A higher index was obtained between GR and PT. The presence of the asterisk indicates significant differences (p-value < 0.001). The numbers in bold indicate the results obtained when the comparative was done within the same population. For this reason, the value is 0 which means that is a single population.

The percentages of polymorphic loci were high in all populations, reaching 100% in CA. The average was 97.49% ± 1.07%. The Shannon index (I) of each population ranged from 0.467 to 0.501. The minimum appeared in PT and the maximum in CA. In this case, no private alleles were found. Finally, all Nm values are very high, which indicates that the populations of study have a strong gene flow among them. An extremely high value was found between CA and SA (35.923), and the lowest was among SA and PT (5.287).

Nei’s genetic distance showed values near 0 (from 0.006 to 0.045), indicating that all *A. pinsapo* nuclei are very similar. However, it is remarkable that GR showed the highest values, which indicates that this population is the most genetically different.

**Figure 4:**
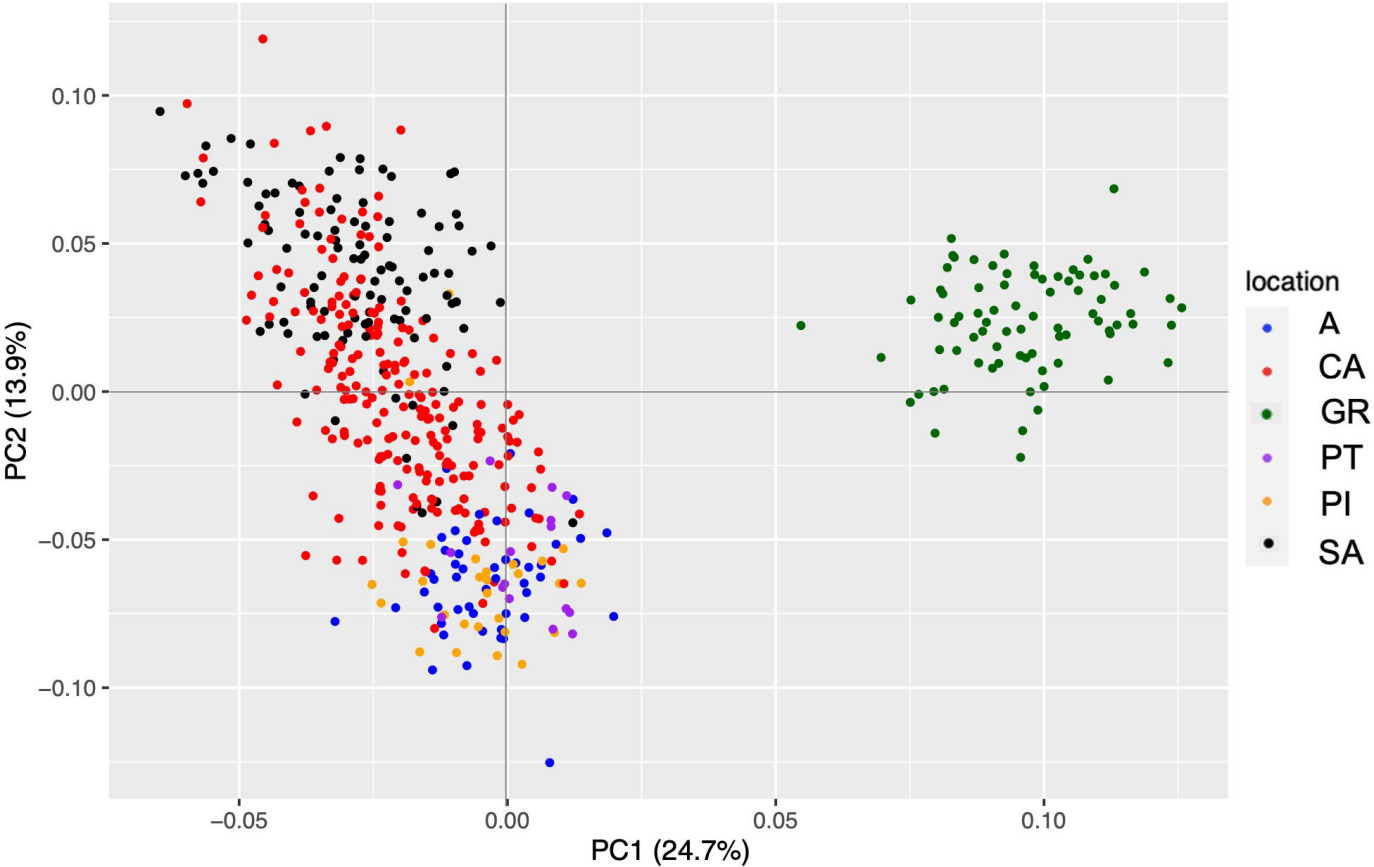
PCA representation of the genetic matrix obtained from the reference assembly with *Abies pinsapo* populations. The meaning of the colors is indicated in the legend. PCA, principal component analysis.

The Spanish fir dataset from the reference assembly filtering retained 494 individuals and 1,642 SNPs. PCA results showed that GR separated from the rest of the populations with an explanation percentage of 24.7% (**Figure 4**). The cross-entropy study, which was carried out with a range from 1 to 8, did not provide much information as in the case of the *de novo* assembly. The admixture results were interpreted by the PCA results, and K = 3 was the best value to explain the genetic structure of our populations. In this case, GR had its own gene pool, SA and CA conformed to other groups, and the rest of the populations were grouped. However, there are several genetic differences among populations as indicated above.

Predictably, the statistical analyses showed the same results, which were obtained with the *de novo* assembly dataset. However, the Nm value between CA and SA was higher (42.561) than the estimated value with the *de novo* assembly. In addition to Nei’s genetic distance ranging from 0.005 to 0.037, the higher value of the range was lower than the estimated value with the *de novo* assembly dataset.

### 3.2 Selection signatures

BayeScan analysis with the *de novo* assembly of *A. pinsapo* nuclei showed 51 loci under selection (q-value < 5%). The complete sequence of the 10 SNPs, which had the lowest q-values, and those that showed associations with bioclimatic variables in GEA studies were queried against the databases. Homolog sequences were found for nine of these sequences, and six of them obtained protein hits (**Table S1**). It is remarkable that two of the used scaffolds gave the same result for both the nucleotide sequence and the proteins. A protein whose function is involved in the dark reaction photosynthesis was obtained (**Table S1**). However, the most interesting proteins found are those that are related to abiotic stress response such as dehydration-responsive element binding transcription factor and the late embryogenesis abundant protein (LEA3-1), which is produced during the seed embryogenesis (**Table S1**).

The Spanish fir reference dataset showed 24 loci under selection. The 10 complete sequences analyzed matched sequences in the NCBI database. However, only four of them obtained hits with proteins (**Table S2**). Two proteins are enzymes involved in photosynthesis and the Calvin cycle. Another homology is found with serine/arginine-rich splicing factor SR45, which is related to the regulation of abscisic acid (ABA) signaling and methylation pathway (**Table S2**).

### 3.3 Genotype–environment associations

The genetic matrix of the *A. pinsapo* populations assembly with the *de novo* approach was used to perform a GEA study. First, after calculating Pearson’s correlation, only those bioclimatic variables that did not show high correlations were used to perform the association study, which were as follows: isothermality (BIO3), maximum temperature of the warmest month (BIO5), temperature annual range (BIO7), precipitation of wettest quarter (BIO16), and precipitation of driest quarter (BIO17). A total of 15 associations with 11 different loci were observed, with the most numerous at the maximum temperature of the warmest month (BIO5) with five loci. Some associations were shared between two or more bioclimatic variables.

**Figure 5:**
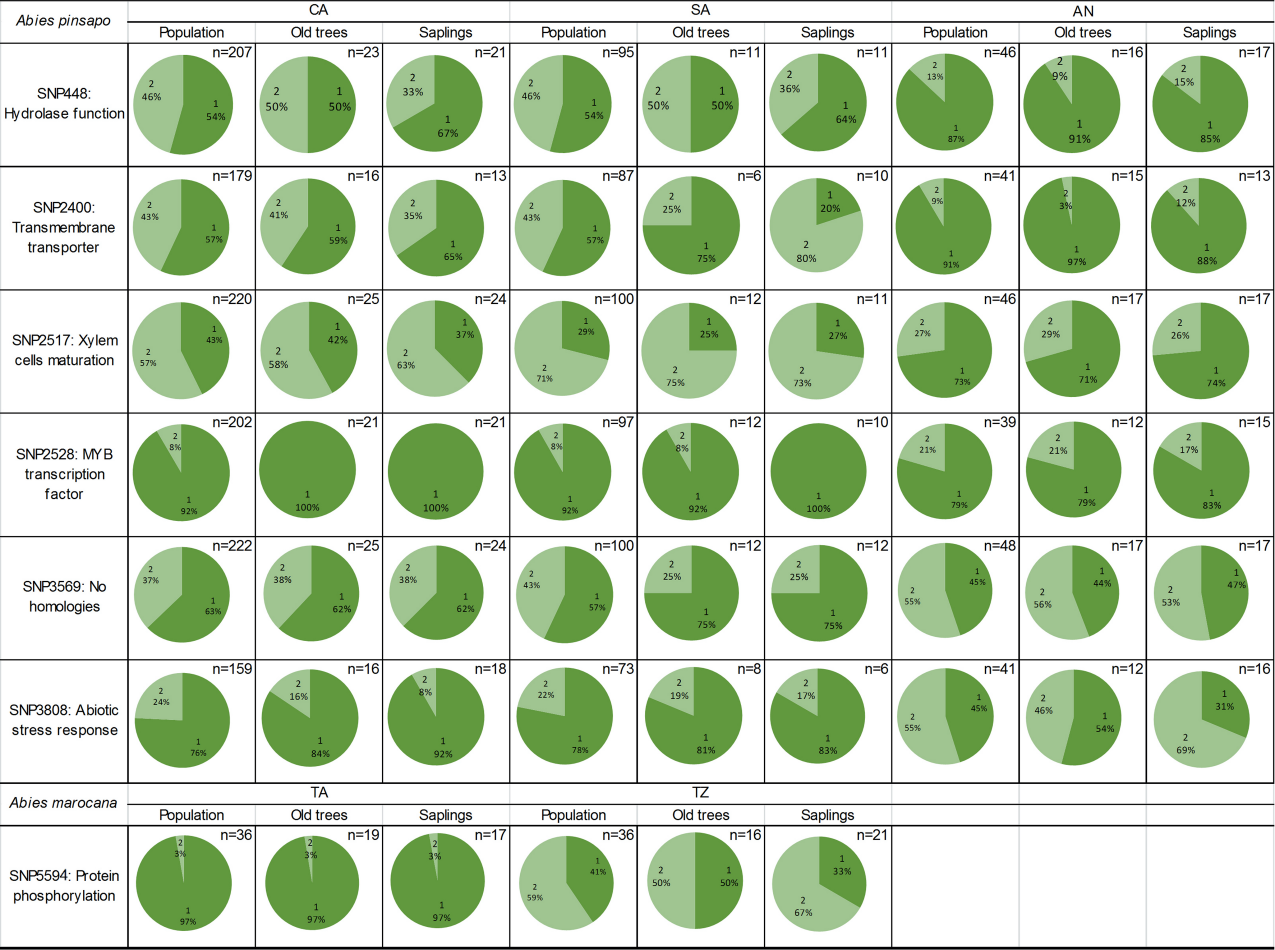
Frequencies of reference allele (1) and alternative allele (2) in SNPs associated with bioclimatic variables in the GEA study of *Abies pinsapo* (CA, SA, and AN populations) and *Abies marocana* (TA and TZ populations). Allele frequencies are shown for the whole population, old trees, and saplings. SNPs, single-nucleotide polymorphisms; GEA, genotype–environment association.

Once the alignments were performed, nine sequences matched nucleotide sequences, and six of them gave us information about homologies with other proteins. Some of these found proteins are related to transmembrane transport, transcription regulation, or abiotic stress response (**Table S3**). Six of these loci were under selection including the locus involved in abiotic stress response. The allele frequencies of some loci were calculated for three *A. pinsapo* populations, which had the two age cohorts (adults and saplings), as follows: CA, SA, and AN. The results indicated that CA and SA populations are very similar to each other, while they are different from AN. The allele frequencies estimated by separating the individuals of these populations into old trees and saplings were slightly different within the CA and SA populations for SNP 448, within the SA population for SNP 2400 and SNP 2528, and within the AN population for SNP 3808 (**Figure 5**).

The GEA study with the reference assembly carried out with *A. pinsapo* populations’ genetic matrix showed 12 associations. The bioclimatic variable called isothermality (BIO3) did not show associations with any locus. This study was performed with the same five bioclimatic variables, which were used with the *de novo* matrix. The highest number of associations was observed with BIO7 (seven loci). The complete sequences of all these loci hit against the nucleotide database, but only five of them matched proteins. Some of their functions were involved in ubiquitination, transmembrane transport, or stomatal closure (**Table S4**).

The GEA study with Moroccan fir populations was carried out with the 19 bioclimatic variables since Pearson’s correlation could not be performed because this genetic matrix had only two populations. The same locus (SNP 5594) was associated with all the variables, and the alignment results showed homology with a protein whose function is related to protein phosphorylation (LRK1). Méndez-Cea et al. (2023b) identified this locus under selection too, so the allele frequencies of each nucleus were calculated. TA had an allele frequency of the reference variant of 0.97, while TZ showed 0.41 for the same allele. When the two sites were separated by age, the saplings from TZ showed a higher allele frequency of the alternative one (0.67) than the adults (0.5), while TA did not show differences for this locus between age groups (**Figure 5**).

### 3.4 Risk of non-adaptedness

The obtained allele frequency change rate value in the low emission scenario for *A. pinsapo* nuclei ranged from 0.01 to 0.47, while in the high emission scenario, the range was 0.02–0.97. In the case of Moroccan fir, the change rate value was between almost 0 to 0.39 for the low emission expected scenario, ranging from approximately 0.02 to 0.90. Considering these results, the high emission scenario showed a higher risk for the Spanish and Moroccan firs’ survival than the other scenario, as expected.

**Figure 6:**
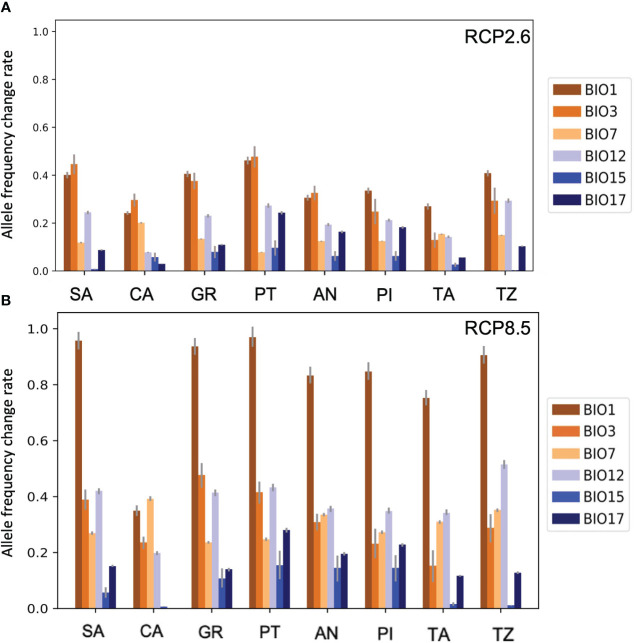
RONA results. Change rate values of each Spanish and Moroccan fir population studied for the low emission scenario **(A)** and the high emission scenario **(B)**. The bioclimatic variables related to temperature are colored in orange, and those related to precipitation appear in blue. The x-axis indicates the nucleus of study, and the y-axis indicates the allele frequency change rate required to respond to the perturbations in each studied variable. RONA, risk of non-adaptedness.

The isothermality (BIO3) showed the maximum change rates for each of the Spanish fir populations in the low emission scenario except for GR and PI populations, which displayed high values in the temperature annual range (BIO1) (**Figure 6A**). The Moroccan fir populations reached higher rates with temperature annual range (BIO1), like GR and PI. However, the annual precipitation (BIO12) was the bioclimatic variable related to precipitations, which showed the maximum change rate value in both species and scenarios (**Figure 6B**).

In terms of the high emission scenario, *A. pinsapo* and *A. marocana* showed the maximum rates with temperature annual range (BIO1). Depending on the Spanish fir population, the second bioclimatic variable, which needs a higher change rate value, was related to temperature (SA, CA, and PT) or precipitation (PI). The second maximum value obtained with Moroccan fir populations (TA and TZ) was always related to annual range precipitation (BIO12).

## 4 Discussion

The development of new genotyping techniques based on NGS technology, such as the ddRAD-seq used in this work, allows to increase the genomic knowledge regarding tree species, which was poorly investigated previously. The number of SNPs detected by ddRAD-seq varied depending on the molecular marker density in the genome of the study (Shirasawa et al., 2016). The number of SNPs maintained after filtering steps was always higher with the *de novo* assembly than with reference. This could be explained by the representation of those SNPs in the reads obtained. Due to the large-size genome of this species (García-García et al., 2022), the likelihood of obtaining the same fragment in most of the individuals sequenced is low. Hence, SNPs are likely to be less represented, and they cannot be retained.

The lack of a reference genome but the availability of Spanish fir transcriptomes gave us the chance to perform two different assembly approaches. Using a transcriptome as a reference allowed us to obtain functional information since all the SNPs described are placed in genes (Card et al., 2014). However, the absence of a correct annotation prevents us to know in some cases the putative biological function of the regions of interest.

Regarding the genetic differences observed among populations, F_ST_ values over 0.15 are usually interpreted as an indicator of significant divergences (Frankham et al., 2002). Hence, considering the low F_ST_ values obtained (<0.15), the six *A. pinsapo* populations might behave as in complete panmixia. Nevertheless, the p-values were significant, indicating that these nuclei showed differences among them. In the light of our results, the locality of GR seems to be the most dissimilar one. Indeed, previous studies carried out with cpSSRs showed differences between GR and Sierra de las Nieves (Terrab et al., 2007; Sánchez-Robles et al., 2012; Cobo-Simón et al., 2020), which is congruent with the present work.

Despite that all the study sites were characterized by a Mediterranean mountain climate, with a summer drought period, the local climate is influenced by elevation and longitude (distance to the Atlantic Ocean). Thus, the mean annual temperature decreases by an average of 0.72°C for every 100 m of elevation, while annual rainfall decreased toward the east by an average of 173 mm for every 10 km and increased by an average of 81 mm for every 100 m of elevation (Linares et al., 2011). As a result, most of the annual rainfall, carried by low-pressure systems coming from Atlantic depressions, falls on the western part of the study area and decreases toward the eastern part, such that high mean precipitation values occur in the westernmost population of Grazalema (GR), as compared to the easternmost population of Sierra de las Nieves (**Figure 1**). This longitudinal differentiation was also reflected in the demographic history inferred by nSSR and cpSSR markers (Sánchez-Robles et al., 2012; Cobo-Simón et al., 2020), indicating that potential adaptive divergence of easternmost and westernmost populations should be subject to further research. Hence, the individuals from GR may have their own genetic pool because they do not have to cope with very harsh drought stress conditions (Cobo-Simón et al., 2020). In addition, the low dispersion of the *A. pinsapo* pollen (lower than 3 km) (Arista and Talavera, 1994; Alba-Sánchez et al., 2010) could act as a limiting factor to genetic exchange.

The noticeable dispersion observed in the PCA for the CA population could be explained by the fact that it is a rear edge of *A. pinsapo* distribution. Since the individuals of this population are under limiting climatic conditions, drier and hotter than average due to low elevation and eastern location, they should have some genetic pressure to cope with them (Cobo-Simón et al., 2020; Cobo-Simón et al., 2021). This is confirmed by the 100% polymorphic loci found in the CA population and by the Shannon index, which showed the highest value among our dataset.

Regarding the loci under selection obtained with *A. pinsapo* populations, one of them also showed associations in the GEA studies, increasing the robustness of the results and highlighting the possible adaptive relevance of this locus. The putative protein homolog identified for this locus, which was under selection and associated with bioclimatic variables, is a dehydration-responsive element binding transcription factor (DREB). Generally, the expression of DREBs is induced by abiotic stresses, such as extreme temperatures, drought, and salinity. Due to this involvement in abiotic stressors responses, the DREB genes of several economically important organisms have been modified with the aim to increase their resistance, such as freezing tolerance in potatoes (i.e., Behnam et al., 2007) and dehydration tolerance in soybean (i.e., de Paiva Rolla et al., 2014) or rice (i.e., Zhao et al., 2010). In terms of studies with angiosperms, Chen et al. (2009) have described the role of DREB genes involved in the responses to cold, drought, and salt in *Populus euphratica* Olivier. Regarding conifers, the involvement of DREB in the drought stress response of *Picea abies* (L.) Karst (Haas et al., 2020) has been reported. Therefore, this locus might be promising for future studies.

Since temperature increase and extreme drought events have been reported in the range of *A. pinsapo* and *A. marocana* (Linares et al., 2009; IPCC, 2022; Méndez-Cea et al., 2023b), the GEA results provide insights into the genetic response of these relict species to the ongoing climate change. *A. pinsapo* populations showed associations with temperature and precipitation variables, indicating that both are important to its development (Alba-Sánchez et al., 2010; López-Tirado and Hidalgo, 2014), while recent studies performed on *A. marocana* pointed out to the main role of temperature (Alaoui et al., 2021; Méndez-Cea et al., 2023b).

Drought events are causing widespread tree mortality across many forest biomes with profound effects on the ecosystem dynamics and carbon balance (Anderegg et al., 2013; McDowell et al., 2020). Our results suggest that the effect of future droughts will almost certainly be worsened by increases in air temperature associated with global warming (**Figure 6**).

The results obtained by comparing saplings and old trees gave a higher number of loci under selection in these two age groups than within the whole population. These contrasting selection signatures may be reflecting the fact that the studied young trees could have been subjected to a selective pressure, as they have been established under a warmer and dryer climate scenario, compared to old trees, which were mainly established around the 19th century. Old trees play key roles in forests and can be disproportionately important to ecosystem carbon storage (McDowell et al., 2020). However, the saplings’ cohorts depict the forest of the future, including the coming allele frequency structure (**Figure 5**). Droughts may have a more detrimental impact on the growth and mortality of old, usually larger, trees (DeSoto et al., 2020). This pronounced drought sensitivity of larger trees might be related to greater inherent vulnerability to hydraulic failure and the higher evaporative demand experienced by their towering crowns (Bennett et al., 2015). Assuming that future extreme drought events, whose frequency is expected to worsen, will have a more detrimental impact on the growth and mortality of old trees, it should be expected to exacerbate feedback to climate change, affecting forest biodiversity and ecosystem services (Anderegg et al., 2013; McDowell et al., 2020).

Reducing uncertainty about tree vulnerability and mortality projections should be founded on robust physiological processes (Anderegg et al., 2012). However, the proposed mechanisms of drought-induced mortality, including hydraulic failure and carbon starvation, are still unresolved (Choat et al., 2018). To that extent, some protein homologies obtained here may provide useful insights into the physiological processes underlying drought-induced tree mortality. One of the homology proteins identified with the *de novo A. pinsapo* dataset in the GEA studies was a transcription factor of the MYB family. This is one of the most relevant and abundant families responsible for plant abiotic stress response/tolerance, and they are often implied in the ABA response, so much so that there are studies that have reported gene expression changes of these transcription factors family during drought stress response in conifer species (Lorenz et al., 2011; Du et al., 2018; de María et al, 2020).

The protein related to stomatal closure, which showed an association between the reference Spanish fir dataset and temperature annual range (BIO7), is called aluminum-activated malate transporter 12-like. In *Arabidopsis thaliana* (L.) Heynh., it is expressed in the guard cells, and as a response to drought stress, this protein induced the stomatal closure as a response to ABA (Meyer et al., 2010). This function is very interesting because most of the gymnosperms, such as *A. pinsapo*, which is an isohydric species, close their stomata to avoid water loss in dry conditions (Brodribb et al., 2014).

In the case of Moroccan fir, the identification of only one locus associated with all the bioclimatic variables, which is also under selection, gave us an insight into the relevance that its genomic region could have. The allele frequency variations found among populations and between ages could indicate that the selection pressure is acting. In addition, a protein homology was identified with a leucine-rich repeat serine/threonine-protein kinase (LRK1). This protein kinase family can be involved in the abiotic stress response (Shiu and Bleecker, 2003). For this reason, this region could be an interesting target for future genotyping in Moroccan fir populations.

Finally, the RONA studies allowed us to describe the genetic vulnerability of the species under future scenarios. Simulating the local adaptation at the population level and their adaptative potential to future conditions is very useful to determine the risk of the species. The use of this kind of study, such as RONA, allows us to integrate the current and future environment data with genetic information to obtain a robust prediction of species adaptation (Feng and Du, 2022). Conifers, as Spanish and Moroccan firs, are long-lived species with long generation times. These characteristics cause them to show a lag in their adaptation to current and future environmental perturbations (Browne et al., 2019). Therefore, the faster climate change is, the greater adaptation lag occurs (Jump and Peñuelas, 2005). Previous studies suggested that conifer species may have difficulty coping with rapid climate change (Dauphin et al., 2021).

Despite recent observational, experimental, and modeling studies suggesting increased vulnerability of relict trees to hotter drought, to our knowledge, this is the first estimation of climate change-induced vulnerability based on genetics and future climate scenarios in *A. pinsapo* and *A. marocana*. RONA modeling supports that warming might be the main limiting factor for the survival of *A. pinsapo* and *A. marocana*, according to several studies performed on drought-sensitive species (e.g., Williams et al., 2013; Allen et al., 2015). Our results support that *A. pinsapo* and *A. marocana* should overcome the high risk of non-adaptedness under any warming climate scenario (**Figure 6**). Previous studies in trees showed that if the change values were less than 0.1 per decade, the species might cope with the climate alterations, while if these changes were higher than the range from 0.1 to 0.2 per decade, the species showed a lag between allele frequencies and environment perturbations (Jump et al., 2006). Dauphin et al. (2021) estimated that the allele frequency variation rate of the conifer species *Pinus cembra* L. was 1.23 × 10^−2^ per generation. Furthermore, the maximum RONA value obtained with *P. cembra* was 0.10 (Dauphin et al., 2021), which is considerably lower than the maximum values estimated for Spanish fir and Moroccan fir. For this reason, both species studied here are likely to have several difficulties coping with the environmental alterations (Esteban et al., 2010; but see also Cortés-Molino et al., 2023). Interestingly, the Moroccan fir population from TZ, with a drier climate, showed the highest vulnerability to variations in annual precipitation at both emission scenarios, suggesting some relationship between the local climate and genetic structure.

Similar results were obtained by dendrochronological studies and climate growth for the whole circum-Mediterranean fir group (Sánchez-Salguero et al., 2017; Méndez-Cea et al., 2023b). Regarding *A. marocana*, previous studies aimed at the potential distribution of Moroccan fir, based on species distribution models (SDMs), showed that the main variables conditioning the presence of *A. marocana* were the average temperature of the warmest quarter and the maximum temperature of the warmest month (Alaoui et al., 2021). Further, similar methodological approaches performed for *A. pinsapo* also obtained a significant effect on temperatures (Alba-Sánchez et al., 2010; López-Tirado and Hidalgo, 2014). Notwithstanding, it is noteworthy that these SDMs are reported as the most important bioclimatic variable driving the modern distribution of *A. pinsapo* winter rainfall, instead of temperature. This disagreement shows that the interpretation of the vulnerability of the species is not straightforward. Temperature (that is, global warming) could change our expectations based on niche suitability and future range distribution of endangered species, such as the circum-Mediterranean firs (Lerner et al., 2023).

Understanding the ecological and evolutionary processes driving the species’ range limits is of particular interest for relict trees owing to their limited distribution and long-term genetic isolation (Hampe and Jump, 2011). Recent studies investigating the underlying forces limiting the range of several tree species support an association of range-edge hotspots with steep spatial climatic gradients (Lerner et al., 2023). Both Spanish and Moroccan firs have a limited distribution, strongly linked to the spatial heterogeneity of climate micro-refugia (Linares, 2011). Thus, the vulnerability of relict trees, as well as those of some isolated range-limit populations, might be hindered because of steep climatic gradients. Further, we present here likely genetic vulnerability drivers, which were, to date, scarcely known. Overall, we obtained estimations of change in allele frequencies higher than 0.1, so both species are at risk because they might not keep pace with climate change due to their change rates being slower than those of the expected environmental shift. The RONA values obtained for *Quercus suber* L. at a high emission scenario (RCP8.5) ranged from 0.07 to 0.38 (Pina-Martins et al., 2019). The estimated values for both Spanish and Moroccan firs in our study at the RCP8.5 scenario (0.97) were higher than the ones in *Q. suber*. Hence, these species are apparently more vulnerable to climate change.

Additional methodological approaches whose relevance is increasing are based, for instance, on machine learning as a promising way to assess the vulnerability of populations in forest species (Ellis et al., 2012). This technology allows us to make predictions at different spatial–temporal scales and can estimate the individual response of SNPs to different environmental variations (Fitzpatrick and Keller, 2015). Furthermore, the development of new genotyping technologies based on NGS that reduce the genome complexity, such as ddRAD-seq, permits to perform genetic studies in conifer species. However, the use of a transcriptome as a reference may give the opportunity to identify SNPs located in genes. In terms of the genetic structure, the *A. pinsapo* populations have shown that all the nuclei behave as in complete panmixia, with existing gene flow among them (see also Sánchez-Robles et al., 2012; Cobo-Simón et al., 2020). Despite this, GR has its own genetic pool that differentiates it from the rest of the populations. GEA results indicated that temperatures and precipitations have effects on both species.

It is widely assumed that warming will lead to hotter droughts and non-linear increasing atmospheric moisture demand. As a result, tree mortality may occur faster as stressful water shortages become more frequent, according to eco-physiological observations and modeling (Linares et al., 2012; Sánchez-Salguero et al., 2015; Choat et al., 2018). As a result, understanding the genetic basis driving the contrasting vulnerability observed among individuals is mandatory. In this context, some studies have described gene expression differences between individuals under drought stress in Mediterranean conifers such as *A. pinsapo* and *Cedrus atlantica* (Endl.) Manetti ex Carrière (Cobo-Simón et al., 2023a; Cobo-Simón et al., 2023b) and in *Pinus halepensis* Mill. (Fox et al., 2018). However, additional studies focused on temperature are still needed. In this sense, our results highlight urgent challenges for research, management, and policy-making communities. The combined analysis of genome-wide single-nucleotide polymorphisms and local environmental data provided genetically based evidence of tree-level sensitivity to climate, estimations of the risk of non-adaptedness, and preliminary evidence of shifting allele frequencies in young cohorts. Notwithstanding, the molecular mechanisms of adaptation remain largely unknown for long-lived tree species. Nonetheless, *A. pinsapo* and *A. marocana* showed high vulnerability to, mainly, temperature variations. The RONA value estimation indicated that both species are at risk because they might not keep pace with climate change. Deciphering such patterns will enable a proper understanding of how endangered tree species are responding to the current changing climate, as well as provide guidelines for future conservation strategies.

## Data availability statement

The raw data presented in the study are deposited in the NCBI’s Sequence Read Archive (SRA) repository, BioProject accesion number PRJNA1000741 (http://www.ncbi.nlm.nih.gov/bioproject/1000741).

## Author contributions

BM-C: investigation, formal analysis, writing—original draft, and writing—review and editing. IG-G: investigation and writing—review and editing. JCL: conceptualization, funding acquisition, formal analysis, and writing—review and editing. FJG: conceptualization, funding acquisition, and writing—review and editing. All authors contributed to the article and approved the submitted version.
