# Supplementary material for: Warming appears as the main risk of non-adaptedness for western Mediterranean relict fir forests under expected climate change scenarios
Source: Front Plant Sci. 2023 Aug 11;14:1155441. doi: 10.3389/fpls.2023.1155441 (PMC10451094; doi:10.3389/fpls.2023.1155441)
Supplement: Supplementary file 1 [file Table_1.docx]

Supplementary Material

Warming appears as the main risk of non-adaptedness for western Mediterranean relict fir-forests under expected climate change scenarios

**Belén Méndez-Cea^1^, Isabel García-García^1^, Juan C. Linares^2^, Francisco Javier Gallego^1*^**

*** Correspondence:** Francisco Javier Gallego fjgalleg@ucm.es

**Table S1:** Protein homologies obtained using the BayeScan results of the *de novo* assembly of *A. pinsapo* populations dataset. It is shown the sequence type, the name and functions of the proteins, and the E-value obtained for each hit (the closer the values are to 0, the greater the probability that the protein function similarity is correct). Protein functions were obtained from UniProt.

| SNP identification | NCBI database | Sequence type | Protein name | Protein function | E-value |
| --- | --- | --- | --- | --- | --- |
| 448 | Nucleotide | mRNA *Picea glauca* | Apyrase 2-like | ATP binding  Hydrolase function | 6e-109 |
| 922 | Nucleotide | mRNA *Picea glauca* | Thermospermine synthase | Auxin polar transport  Phloem and xylem histogenesis  Xylem vessel member cell differentiation | 2e-38 |
| 1563 | TSA | Transcribed RNA sequence of *Pseudotsuga menziesii* | Heavy metal-associated isoprenylated plant protein 45 | Heavy metal detoxification (Cd)  Transcriptional response to cold and drought  Plant-pathogen interactions | 3e-26 |
| 2322 | Nucleotide | mRNA *Picea sitchensis* | Light-independent protochlorophyllide reductase subunit N | Chlorophyllide synthesis  Dark reaction of photosynthesis | 5e-65 |
| 2400 | Nucleotide | mRNA *Picea sitchensis* | D-xylose-proton symporter-like3  (chloroplast) | Transmembrane transporter | 0.0 |
| 2517 | Nucleotide | mRNA *Picea glauca* | Thermospermine synthase | Auxin polar transport  Phloem and xylem histogenesis  Xylem vessel member cell differentiation | 2e-38 |
| 2528 | Nucleotide | mRNA *Nelumbo nucifera* | (Predicted) Transcription factor MYB39-like | Suberin biosynthesis  Regulation of transcription | 6e-169 |
| 2700 | Nucleotide | mRNA *Picea sitchensis* | Late embryogenesis abundant protein (LEA3-1) | Stress tolerance responses (drought) | 2e-50 |
| 3808 | Nucleotide | mRNA *Picea sitchensis* | Dehydration-responsive element binding transcription factor | Abiotic stress response | 4e-58 |

**Table S2**: Protein homologies obtained using the BayeScan results of reference assembly *A. pinsapo* populations dataset. It is shown the sequence type, the name and functions of the proteins, and the E-value obtained for each hit (the closer the values are to 0, the greater the probability that the protein function similarity is correct). Protein functions were obtained from UniProt.

| SNP identification | NCBI database | Sequence type | Protein name | Protein function | E-value |
| --- | --- | --- | --- | --- | --- |
| 325 | TSA | Transcribed RNA sequence *Abies pinsapo* | Phosphoenolpyruvate carboxylase | Convert phosphoenolpyruvate and bicarbonate into oxaloacetate and inorganic phosphorus  Photosynthesis | 1e-31 |
| 370 | Nucleotide | mRNA *Cucumis melo* | WRKY transcription factor 9 | Defense response | 0.0 |
| 974 | Nucleotide | Chloroplast genome of *Abies alba* | Ribulose-1,5-bisphosphate carboxylase/oxygenase large subunit (RUBISCO) | Calvin cycle: dioxide carbon fixation | 4e-111 |
| 1313 | Nucleotide | mRNA *Picea sitchensis* | Serine/arginine-rich splicing factor SR45 | RNA-directed DNA methylation pathway  Splicing site selection of introns  Negatively regulation of ABA signaling | 6e-52 |

**Table S3**: Results of GEA study with the genetic matrix of *A. pinsapo* populations obtained in the *de novo* assembly. It is shown the sequence type, the name and functions of the proteins, and the E-value obtained for each hit (the closer the values are to 0, the greater the probability that the protein function similarity is correct). Protein functions were obtained from UniProt.

| SNP identification | NCBI database | Sequence type | Protein name | Protein function | E-value |
| --- | --- | --- | --- | --- | --- |
| 448 | Nucleotide | mRNA *Picea glauca* | Apyrase 2-like | ATP binding  Hydrolase function | 6e-109 |
| 2400 | Nucleotide | mRNA *Picea sitchensis* | D-xylose-proton symporter-like3  (chloroplast) | Transmembrane transporter | 0.0 |
| 2517 | Nucleotide | mRNA *Picea glauca* | Thermospermine synthase | Auxin polar transport  Phloem and xylem histogenesis  Xylem vessel member cell differentiation | 2e-38 |
| 2528 | Nucleotide | mRNA *Nelumbo nucifera* | (Predicted) Transcription factor MYB39-like | Suberin biosynthesis  Regulation of transcription | 6e-169 |
| 3808 | Nucleotide | mRNA *Picea sitchensis* | Dehydration-responsive element binding transcription factor. | Abiotic stress response | 4e-58 |
| 3961 | Nucleotide | mRNA *Larix sibirica* | DDE-type integrase/transposase/recombinase | DNA integration | 1e-08 |

**Table S4:** GEA results obtained with *A. pinsapo* populations dataset from the reference assembly. It is shown the sequence type, the name and functions of the proteins, and the E-value obtained for each hit (the closer the values are to 0, the greater the probability that the protein function similarity is correct). Protein functions were obtained from UniProt.

| SNP identification | NCBI database | Sequence type | Protein name | Protein function | E-value |
| --- | --- | --- | --- | --- | --- |
| 249 | Nucleotide | mRNA *Picea glauca* | E3 ubiquitin-protein ligase | Promotion of protein ubiquitination and degradation | 6e-139 |
| 691 | Nucleotide | mRNA *Picea sitchensis* | Class I chitinase | Hydrolyzation of N-acetylglucosamine polymer chitin | 1e-133 |
| 697 | Nucleotide | mRNA *Picea glauca* | NEDD8-activating enzyme E1 regulatory subunit | Ubiquitination | 5e-125 |
| 872 | Nucleotide | mRNA *Picea sitchensis* | D-xylose-proton symporter-like 3 (chloroplast) | Transmembrane transport | 0.0 |
| 1609 | TSA | Transcribed RNA of *Pinus patula* | Aluminum-activated malate transporter 12-like | Stomatal closure induced by dark, ABA, water deficient | 5e-120 |
